# Treatment of Epistaxis by Internal Administration of Ergot

**Published:** 1876-08

**Authors:** 


Treatment of Epistaxis by the Internal Administration of Ergot.—Dr. George St. George, of Lisbon, observes that the treatment of epistaxis is often attended with great difficulty, especially in persons enfeebled by age. He has found ergot of use in cases where liquor ferri perchloridi, plugging, and other remedies have been tried without avail. The following is one of the cases he records. A weak anæmic woman aged 55, had been suffering for three days from repeated and violent attacks of hemmorrhage from the nose, which had increased in both frequency and violence during the last twenty-four hours. The nostrils were first plugged with lint and cold water. Dr. St. George then tried plugging with lint dipped in liquor ferri perchloridi, but without avail, as the hæmorrhage continued. He then ordered her a mixture, each dose to contain fifteen minims, of liquid extract of ergot every quarter of an hour until the hæmorrhage ceased, and then to be continued every four hours for a day or two. In an hour and a half the bleeding had entirely ceased, and never returned. He gives the details of two other cases successfully treated by the same means. —*British Medical Journal.*

